# Cerebral amebiasis due to *Acanthamoeba* sp. in a patient with complete gp91^*phox*^ deficiency

**DOI:** 10.70962/jhi.20250160

**Published:** 2026-03-10

**Authors:** Marie Roelens, Anna-Lena Neehus, Jérémie Rosain, Gislène Collobert, Nathalie Boddaert, Liana Carausu, Jacinta Bustamante

**Affiliations:** 1 Study Center for Primary Immunodeficiencies, Necker Hospital for Sick Children, Assistance Publique-Hôpitaux de Paris (AP-HP), Paris, France; 2 Paris Cité University, Imagine Institute, INSERM, U1163, Paris, France; 3 Immunology Laboratory, Necker Hospital for Sick Children, AP-HP, Paris, France; 4 Laboratory of Human Genetics of Infectious Diseases, Necker Branch, Paris, France; 5 Division of Hematology/Oncology, Boston Children’s Hospital and Department of Pediatric Oncology, Dana-Farber Cancer Institute, Harvard Medical School, Boston, MA, USA; 6 St. Giles Laboratory of Human Genetics of Infectious Diseases, Rockefeller Branch, The Rockefeller University, New York, NY, USA; 7 Department of Pediatric Radiology, Necker Hospital for Sick Children, AP-HP, Paris, France; 8 Department of Pediatrics, Morvan Hospital, Brest, France

## Abstract

A patient diagnosed with chronic granulomatous disease due to complete gp91^*phox*^ deficiency developed multiple cerebral abscesses caused by *Acanthamoeba* species. This case highlights that invasive parasitic infections can reveal underlying phagocytic defects and underscores for prompt immunological and genetic evaluation in such situations.

## Teaser

Rare variants in *CYBB* (encoding gp91^*phox*^) are responsible for chronic granulomatous disease. We report a patient affected by multiple cerebral abscesses caused by *Acanthamoeba sp*. and complete gp91^*phox*^ deficiency. The respiratory burst evaluation is recommended in any child with a deep abscess.

Chronic granulomatous disease (CGD) is an inborn error of immunity (IEI) caused by rare variants affecting any of the five genes encoding nicotinamide adenine dinucleotide phosphate oxidase subunits (*CYBB, CYBA, NCF1, NCF2,* or *NCF4*) or the EROS chaperone (*CYBC1*) (1). These variants result in an absence (for classic CGD) or impairment (for variant CGD) of the production of superoxide (O_2_^−^) and other reactive oxygen species (ROS) by phagocytic cells, including polymorphonuclear neutrophils (PMNs), eosinophils, monocytes, macrophages, and dendritic cells. CGD has an incidence of 1 in 100,000–200,000 live births. It is more frequent in males, with about two thirds of cases caused by the X-linked recessive inheritance of rare variants of *CYBB*, which encodes the gp91^*phox*^ subunit. CGD patients mostly suffer from severe infectious diseases, but they are also prone to hyperinflammation, leading to granuloma formation in various organs, and inflammatory bowel disease in particular. Pyogenic bacterial, mycobacterial, and fungal infections are frequently reported in these patients, some of whom have a narrow susceptibility to particular pathogens, especially *Aspergillus* sp., *Candida *sp., *Staphylococcus aureus*, *Burkholderia cepacia*, *Serratia marcescens*, *Nocardia* sp., *Salmonella* sp., *Mycobacterium tuberculosis*, and the *Mycobacterium bovis* strain used as the bacille Calmette–Guérin (BCG) vaccine. The skin, lymph nodes, bones, lungs, and deep organs, such as the brain, are common sites of infection. Unlike bacteria and fungi, parasites have rarely been reported to cause disease in CGD cohorts, and invasive or disseminated parasitic infections are even less common (1). We report here the first case of classic CGD due to gp91^*phox*^ deficiency, in a child with multiple infectious brain abscesses due to *Acanthamoeba* sp*.*

**Figure 1.**
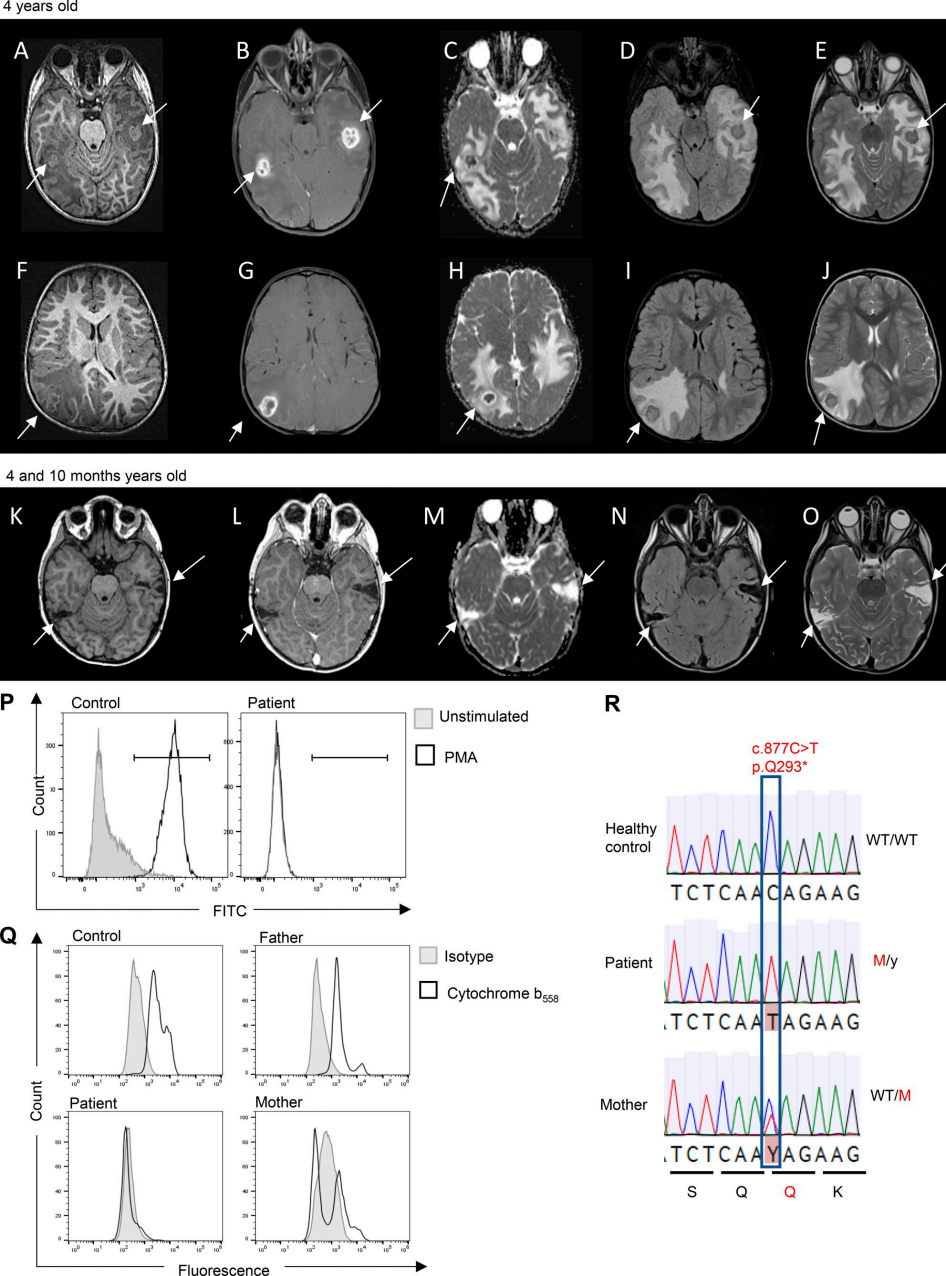
**Parasitic infection in a patient with complete gp91** ^***phox***^ **deficiency.** Left to right: Brain MRI sequences of axial slices in unenhanced T1, T1 with contrast, diffusion ADC (apparent diffusion coefficient), FLAIR, and T2-weighted images. **(A–E)** At the level of the brainstem, the presence of two amebiasis brain abscesses in the temporal lobe/cerebrum with abnormal tissue enhancement on the T1 without injection (A) compared with postcontrast T1-weighted images (B) associated with significant edema all around the abscess and hypointense collections with low ADC (C) suggesting cytotoxic edema and abnormal FLAIR (D) and T2 (E). **(F–J)** Presence of one abscess with the same characteristics in the cerebral cortex: abnormal heterogeneous enhancement on postcontrast T1-weighted images (G) compared to the T1 without injection (F) with hypointense collection with low ADC (H) and an abnormal FLAIR (I) and T2 (J) with significant edema all around the abscess. **(K–O)** Radiological improvement after treatment is demonstrated by arrows. There is no longer any contrast enhancement (L) compared to T1 without injection (K), and the ADC is increased (M). Sequelae of the abscess with cortico-subcortical atrophy on flair (N) and T2 (O) in place of the abscess in the two temporal lobes. **(P)** DHR oxidation test on PMNs from fresh peripheral blood obtained from the healthy control and the patient. 100% of DHR was oxidized after the stimulation of control PMNs with PMA, leading to a fluorescent signal in the FITC channel. The DHR remained nonfluorescent after the stimulation of PMNs from the patient. **(Q)** Cytochrome b_558_ expression on PMNs from a healthy control, the patient, and his relatives (M: c.877C>T; WT: wild-type). **(R)** Electropherogram for the patient, his mother, and a healthy control.

The patient, a boy, was seen in consultation at the age of 4 years. He was born at term and was the first child of a nonconsanguineous Georgian couple, neither of whom had any significant medical history. His early childhood was marked by recurrent otitis, and hospitalization at the age of 4 mo for infectious mononucleosis. He was vaccinated according to the Georgian vaccination schedule, which includes two live vaccines: BCG at birth and rotavirus vaccine at the ages of 4 and 5 mo. At the age of 11 months, the patient developed axillary adenopathy, which was assumed to be linked to the BCG vaccine (BCG-itis) and resolved after antimycobacterial treatment for 2 mo with a combination of rifampicin, isoniazid, and ethambutol, followed by 4 mo of treatment with rifampicin and isoniazid. At the age of 4 years, he was referred to a pediatric unit in Turkey for persistent fever and asthenia with otalgia and otorrhea, despite 3 mo of treatment with appropriately administered oral and local antibiotics. No predisposed factors of immunodeficiency were identified in this patient. Blood leukocyte count (24,000/mm^3^) and C-reactive protein levels (159 mg/l) were high, whereas procalcitonin levels and the results of cerebrospinal fluid examination were normal. A computed tomography scan revealed right mastoiditis associated with otitis, leading to treatment with meropenem and vancomycin. However, 7 days later, the patient suffered partial seizures in his left arm. Magnetic resonance imaging (MRI) of the brain disclosed multiple brain abscesses (Fig. 1, A–J). The patient was transferred to Georgia, where he underwent brain surgery. A biopsy specimen obtained during surgery contained amebic cysts and trophozoites and yielded a positive PCR result for the detection of *Acanthamoeba* sp*.* Cultures of the same sample were negative for other microbes, including pyogenic bacteria, mycobacteria, and viruses. Toxoplasmosis was also excluded by PCR. Keratitis was not documented. A transient improvement of fever was observed after 8 wk of treatment with meropenem, vancomycin, and metronidazole, despite radiological progression. The patient then received a second-line antibiotic treatment with linezolid and voriconazole (due to a positive antigenemia for *Aspergillus*) without clinical improvement. Surgery with the extirpation of the cerebral abscess improved definitively the patient’s clinical state. No new abscesses were identified after surgery. 10 mo after the infectious episode, a control cerebral MRI scan in France showed only scar lesions (Fig. 1, K–O). Anti-epileptic treatment with levetiracetam was maintained for 2 years, and the patient’s final neurological evaluation was normal.

Given the very rare and atypical nature of this infection, an IEI was suspected. 8 mo after this episode of amebiasis, serum immunoglobulin levels were normal—IgG, 8.3 g/l (normal range [NR]: 5.5–10.2 g/l); IgA, 1.51 g/l (NR: 0.41–1.41 g/l); and IgM, 0.97 g/l (NR: 0.5–1.5 g/l)—as were counts of lymphocytes (2,490 lymphocytes/mm^3^, NR: 1,500–7,500/mm^3^), CD4^+^ T cells (875/mm^3^, NR: 500–2,400/mm^3^), CD8^+^ T cells (642/mm^3^, NR: 300–1,600/mm^3^), B cells (549/mm^3^, NR: 200–2,100/mm^3^), and natural killer cells (187/mm^3^ NR: 100-1,000/mm^3^). Naïve and memory T cell subsets were present in proportions appropriate for the patient’s age. However, PMNs from the patient were unable to produce ROS *in vitro* in response to stimulation with phorbol 12-myristate 13-acetate. For the healthy control, 100% of the dihydrorhodamine-1,2,3 (DHR) used in the assay was oxidized after PMN stimulation (Fig. 1 P). This result strongly suggested a diagnosis of classic CGD. Moreover, an abolition of cytochrome b_558_ expression (the membrane-bound gp91^*phox*^ and p22^*phox*^ heterodimer) on PMNs was detected by flow cytometry (Fig. 1 Q). This finding was confirmed by Sanger sequencing, which revealed a previously described hemizygous variant in exon 8 of the *CYBB* gene, c.877C>T (NM_000397.3) (Fig. 1 R). This nucleotide substitution creates a stop codon, p.Gln293*, leading to the production of a truncated nonfunctional protein. The child’s mother was heterozygous for the same mutation, whereas the father was wild-type. Since the documentation of this IEI, the patient has been on continuous prophylaxis (itraconazole [10 mg/kg/day] and cotrimoxazole [25 mg/kg/day]) to protect against infection. No relative with the potential to act as a donor for hematopoietic stem cell transplantation (HSCT) has been identified. However, an HSCT with unrelated donor (adult donor or umbilical cord blood banks) is underway.

We report here the first case of invasive *Acanthamoeba* infection in a patient with CGD, leading to brain abscesses. This patient also developed BCG-itis during his first year of life—a frequent manifestation in CGD patients from countries in which BCG vaccination is obligatory. *Acanthamoeba sp.* is a ubiquitous protozoan—widespread in soil, water, and air—to which most humans become immunized at some point during their lives. The cystic (dormant) form can survive for years in hostile environments, whereas the active amebic trophozoite form needs to feed on organic particles to divide. This protozoan is a frequent cause of keratitis (local corneal infection) in the general population, but invasive forms, such as amebic encephalitis or pneumonia, are far rarer and assumed to result from a passage of the parasite from the mucosa into the bloodstream. Amebic encephalitis is often fatal, notably due to the complexity of its diagnosis, which delays the administration of appropriate treatments. There is no clear consensus concerning treatment, which is mostly based on multidrug regimens including liposomal amphotericin B, azole antifungal agents, pentamidine, azithromycin, sulfadiazine, rifampicin, 5-flucytosine, cotrimoxazole, and miltefosine. This condition occurs mostly in patients with acquired immunodeficiency syndrome or on immunosuppressive treatments. Two cases of amebic encephalitis in patients with underlying extrapulmonary tuberculosis have been described, but these patients did not undergo genetic screening for known IEIs.

Parasitic infections are rarely documented in patients with IEIs, especially in European cohorts. Several common local digestive infections have been described in Central and Latin American CGD cohorts, caused by *Entamoeba histolytica*, *Endolimax nana* (another two amoeba species), *Ascaris lumbricoides*, *Giardia lamblia*, *Cryptosporidium parvum*, or *Toxocara spp*. (1). An invasive infection due to *Echinococcus granulosus* resulting in a liver hydatid cyst has been reported in a Turkish patient with p22^*phox*^ deficiency. Several cases of visceral leishmaniasis have been documented in patients of Mediterranean origin with gp91^*phox*^ or p47^*phox*^ deficiencies and in patients with other inborn errors of interferon gamma (IFN-γ)–mediated immunity (IL-12p40, IL-12Rβ1, or IFN-γR1 deficiencies) or OX40 deficiency (2). Cryptosporidiosis has also been reported in many patients with combined immunodeficiencies (CID), including hyper-IgM syndrome due to CD40 or CD40L deficiency, and CID involving impairment of the NF-κB pathway (3). Finally, disseminated *Toxoplasma gondii* infections have also been observed in several IEIs, but not, to date, in CGD (4). An invasive amebic infection such as that seen in this CGD patient suggests a role of phagocytic cells and their respiratory burst in the control of this parasite. The mechanisms involved are not well known, but studies in mice suggest a role for receptors of innate immunity in response to *Acanthamoeba sp.* infection—especially Toll-like receptors (TLR2, TLR4)—and for specific Th1 lymphocytes, with IFN-γ playing a key role (5). This cytokine is known to stimulate ROS production by phagocytic cells. It has also been shown in mice that protozoan infections elicit the formation of neutrophil extracellular traps, a process that is defective in CGD. Amebic abscesses can reveal CGD, although this is unusual in patients with IEIs of phagocytic cells. Therefore, in the absence of secondary immunosuppression factors, an IEI should be considered in patients with invasive parasitic infections, and in-depth immunological and genetic explorations are justified in such cases, to ensure appropriate medical care and family genetic counseling. Testing of the respiratory burst in a DHR assay is recommended in the evaluation of all children with deep abscesses.
